# Candidate gene–environment interactions and their relationships with timing of breeding in a wild bird population

**DOI:** 10.1002/ece3.1630

**Published:** 2015-08-11

**Authors:** Audrey Bourret, Dany Garant

**Affiliations:** Département de biologie, Université de Sherbrooke2500 boulevard de l'Université, Sherbrooke, QC, J1K 2R1, Canada

**Keywords:** ADCYAP1, candidate gene, CLOCK, CREB1, GxE, incubation duration, laying date, NPAS2

## Abstract

Monitoring and predicting evolutionary changes underlying current environmental modifications are complex challenges. Recent approaches to achieve these objectives include assessing the genetic variation and effects of candidate genes on traits indicating adaptive potential. In birds, for example, short tandem repeat polymorphism at four candidate genes (CLOCK, NPAS2, ADCYAP1, and CREB1) has been linked to variation in phenological traits such as laying date and timing of migration. However, our understanding of their importance as evolutionary predictors is still limited, mainly because the extent of genotype–environment interactions (GxE) related to these genes has yet to be assessed. Here, we studied a population of Tree swallow (*Tachycineta bicolor*) over 4 years in southern Québec (Canada) to assess the relationships between those four candidate genes and two phenological traits related to reproduction (laying date and incubation duration) and also determine the importance of GxE in this system. Our results showed that NPAS2 female genotypes were nonrandomly distributed across the study system and formed a longitudinal cline with longer genotypes located to the east. We observed relationships between length polymorphism at all candidate genes and laying date and/or incubation duration, and most of these relationships were affected by environmental variables (breeding density, latitude, or temperature). In particular, the positive relationships detected between laying date and both CLOCK and NPAS2 female genotypes were variable depending on breeding density. Our results suggest that all four candidate genes potentially affect timing of breeding in birds and that GxE are more prevalent and important than previously reported in this context.

## Introduction

Current environmental changes, such as climate warming, severely impact natural populations by generating new and/or modifying already existing selective pressures (Parmesan 2006; Hendry et al. 2008). To cope with these novel conditions, populations can disperse to more suitable habitats and exhibit phenotypic plasticity and/or an evolutionary adaptive response (Gienapp et al. 2008; Hoffmann and Sgrò 2011; Merilä 2012). Over the long term, a population evolutionary response to selection should involve genetic changes (Hoffmann and Sgrò 2011). However, monitoring and predicting these changes have proved to be challenging. Recent approaches to achieve these objectives in natural populations include assessing the genetic variation and effects of candidate genes on traits indicating adaptive potential in the face of environmental fluctuations (Hoffmann and Willi 2008; Hoffmann and Sgrò 2011; Pardo-Diaz et al. 2015).

A candidate gene approach tests statistical correlations between phenotypes and specific a priori relevant genetic components (i.e., identified/suspected from previous biochemical studies or of known influence in another species) to link phenotypic variations to gene variants (Fitzpatrick et al. 2005; Hoffmann and Willi 2008). More specifically, short tandem repeats (STR) are present in neutral (e.g., microsatellites) and functional genome regions, but it is their variation in repeat numbers within functional genome regions (5′-UTR, exons, introns, 3′-UTR) that may modify gene functions (mainly the level of genic expression, see Elmore et al. 2012) and resulting phenotypes (Kashi et al. 1997; Comings 1998; Li et al. 2004; Fondon et al. 2008) and thus represent potential candidate genes. For example, several STR length polymorphisms are associated with the presence of some human diseases (e.g., Huntington's disease) and variation in animal behaviors (e.g., vasopressin-dependent social behavior in prairie voles *Microtus ochrogaster*; reviewed in Fondon et al. 2008).

Recent studies in birds have highlighted four candidate genes showing STR length polymorphisms associated with phenological traits and thus relevant to study in the context of changing environmental conditions (Johnsen et al. 2007; Steinmeyer et al. 2009; see Table1 for a summary). The most commonly studied gene so far is CLOCK, a highly conserved transmission factor central to the rhythmicity of the circadian oscillator (reviewed in Young and Kay 2001). CLOCK possesses a poly-Q binding region that shows length polymorphism (in Q repeat number) which affects its binding affinity with its transmission factor (Darlington et al. 1998). At the population level, a positive latitudinal gradient in the number of poly-Q repeats has been observed across blue tit populations in Europe (*Cyanistes caeruleus*, Johnsen et al. 2007). This gradient was steeper than expected under neutral processes, thus suggesting an underlying functional basis to the genetic polymorphism (Kyriacou et al. 2008). However, this gradient was not detected in the two other bird species where it was assessed (bluethroats (*Luscinia svecica*), Johnsen et al. 2007; pied flycatchers (*Ficedula hypoleuca*), Kuhn et al. 2013), raising doubts about the generality of this finding. At the individual level, length polymorphism in CLOCK was positively correlated with female laying date, hatching date and incubation duration in blue tits (Liedvogel et al. 2009) and laying date in barn swallows (*Hirundo rustica*, Caprioli et al. 2012). Nevertheless, such relationships were absent for the same traits studied in several other bird species (see Table1). In a common buzzard (*Buteo buteo*) population, STR length polymorphism in three other candidate genes, NPAS2, ADCYAP1 and CREB1, has been recently reported for the first time in relationship to reproduction timing (Chakarov et al. 2013; Table1). The candidate gene NPAS2 shows length polymorphism in the same exon as its paralog CLOCK (Steinmeyer et al. 2009) and is believed to overtake its functions (Debruyne 2008). The two others, the neurotransmitter ADCYAP1 and the transcription factor CREB1, have shown STR polymorphism in their 3′-UTR region (Steinmeyer et al. 2009) and both have a broad spectrum of functions related in part with the circadian rhythm core oscillator (Carlezon et al., 2005; Vaudry et al. 2009). These three candidate genes have not shown significant relationship to reproductive timing in the only population studied (Chakarov et al. 2013). However, relationships between length polymorphism at these genes and other phenological traits, such as dispersal and migration behavior, were reported in different bird species (Mueller et al. 2011; Chakarov et al. 2013; Peterson et al. 2013).

**Table 1:** Summary of individual-based studies assessing relationships between candidate gene polymorphisms (CLOCK, NPAS2, ADCYAP1, and CREB1) and phenotypic variation at phenological traits related to reproduction. Number of years and individuals used (for both sexes if known), the presence of a significant relationship (and the direction if significant) and gene-environment (GxE) interactions (YES: tested and significant; NO: tested and nonsignificant; –: not tested) are reported

Trait	Species	Localization	Gene	*N* years	*N* individuals (F/M)	Relationship (direction)	GxE	Reference
Laying date	Barn swallow (*Hirundo rustica*)	Milano, Italy	CLOCK	4	922 (478/444)	YES (+)	–	Caprioli et al. (2012)
	Blue tit (*Cyanistes caeruleus*)	Wytham Woods, Oxfordshire, UK	CLOCK	2	950 (539/411)	YES (+)	NO	Liedvogel et al. (2009)
	Chilean swallow (*Tachycineta meyeni*)	Ushuaia, Argentina	CLOCK	3	88 (88/–)	NO	–	Dor et al. (2012)
	Great tit (*Parus major*)	Wytham Woods, Oxfordshire, UK	CLOCK	5	521 (521/–)	NO	NO	Liedvogel and Sheldon (2010)
	Mangrove swallow (*Tachycineta albilinea*)	Hill Bank, Belize	CLOCK	3	163 (163/–)	NO	–	Dor et al. (2012)
	Pied flycatcher (*Ficedula hypoleuca*)	La Hiruela, Spain	CLOCK	1	42 (26/16)	NO	–	Kuhn et al. (2013)
	Tree swallow (*Tachycineta bicolor*)	Ithaca, NY, USA	CLOCK	9	548 (548/–)	NO	–	Dor et al. (2012)
	Violet-green swallow (*Tachycineta thalassina*)	Mono Lake, CA, USA	CLOCK	2	48 (48/–)	NO	–	Dor et al. (2012)
	White-rumped swallow (*Tachycineta leucorrhoa*)	Chascomús, Argentina	CLOCK	2	169 (169/–)	NO	–	Dor et al. (2012)
Hatching date	Blue tit (*Cyanistes caeruleus*)	Wytham Woods, Oxfordshire, UK	CLOCK	2	950 (539/411)	YES (+)	NO	Liedvogel et al. (2009)
	Great tit (*Parus major*)	Wytham Woods, Oxfordshire, UK	CLOCK	5	521 (521/–)	NO	NO	Liedvogel and Sheldon (2010)
Incubation duration	Blue tit (*Cyanistes caeruleus*)	Wytham Woods, Oxfordshire, UK	CLOCK	2	950 (539/411)	YES (+)	NO	Liedvogel et al. (2009)
	Great tit (*Parus major*)	Wytham Woods, Oxfordshire, UK	CLOCK	5	521 (521/–)	NO	NO	Liedvogel and Sheldon (2010)
	Mangrove swallow (*Tachycineta albilinea*)	Hill Bank, Belize	CLOCK	3	163 (163/–)	NO	–	Dor et al. (2012)
	Tree swallow (*Tachycineta bicolor*)	Ithaca, NY, USA	CLOCK	9	548 (548/–)	NO	–	Dor et al. (2012)
	Violet-green swallow (*Tachycineta thalassina*)	Mono Lake, CA, USA	CLOCK	2	48 (48/–)	NO	–	Dor et al. (2012)
	White-rumped swallow (*Tachycineta leucorrhoa*)	Chascomús, Argentina	CLOCK	2	169 (169/–)	NO	–	Dor et al. (2012)
Timing of broods[Table-fn tf1-1]	Common buzzard (*Buteo buteo*)	Eastern Westphalia, Germany	CLOCK	11	479[Table-fn tf1-2]	–[Table-fn tf1-3]	–	Chakarov et al. (2013)
			NPAS2	11	479[Table-fn tf1-2]	NO		
			ADCYAP1	11	479[Table-fn tf1-2]	NO	–	
			CREB1	11	479[Table-fn tf1-2]	NO	–	

Timing of broods reflects timing of fledglings within a brood compared to the timing of fledglings in other broods within the same year.

Genotypes were defined as the average of nestling genotypes within a nest (*N* = 976), thus reflecting both male and female genotypes.

CLOCK was monomorphic, and thus, no further analysis was made.

Despite the potential of these candidate genes to reflect, at least partially, the genetic basis of phenological traits related to reproduction, our understanding of their importance in natural population is still limited for several reasons. First, apart from the study by Chakarov et al. (2013) on common buzzards, the four candidate genes have rarely been studied in the same population. Second, there are important discrepancies among studies in term of sample sizes, reducing the detection probability of small to intermediate gene effect sizes (Manolio et al. 2009). Also, previous studies generally focussed on female-specific analyses despite the potential importance of male genetic effects on phenological traits (e.g., Teplitsky et al. 2010). Finally and importantly, despite some evidences of gene–environment interactions (i.e., GxE) being present across populations, very few studies assessed GxE within a population (but see Liedvogel et al. 2009 and Liedvogel and Sheldon 2010). At the individual level, the presence of GxE could explain the lack of relationships between candidate genes and phenological traits documented in previous studies.

Here, we used 4 years of data from a Tree swallow (*Tachycineta bicolor*) long-term study in southern Québec (Canada) to investigate the relationship between length polymorphism at all four candidate genes (CLOCK, NPAS2, ADCYAP1, and CREB1) and phenological traits related to reproduction (laying date and incubation duration). Tree swallow is a small migratory passerine, and while laying date for this species has advanced in North America during the last decades (Dunn and Winkler 1999; Rioux Paquette et al. 2014), we still know little about the underlying genetic basis of this trait. For example, a single study in this species analyzed CLOCK variation in females within a population based in Ithaca (NY, USA) – no relationships were found between length polymorphism and laying date or incubation duration (Dor et al. 2012). In this study, our objectives were to (1) describe variation at the four candidate genes in the southern Québec population, (2) assess the geographic and environmental variations in the genes, and (3) examine the relationships between variation at these genes for both males and females and phenotypic variation in laying date and incubation duration, while determining the importance of GxE in this system.

## Methods

### Study system and data collection

**Figure 1:**
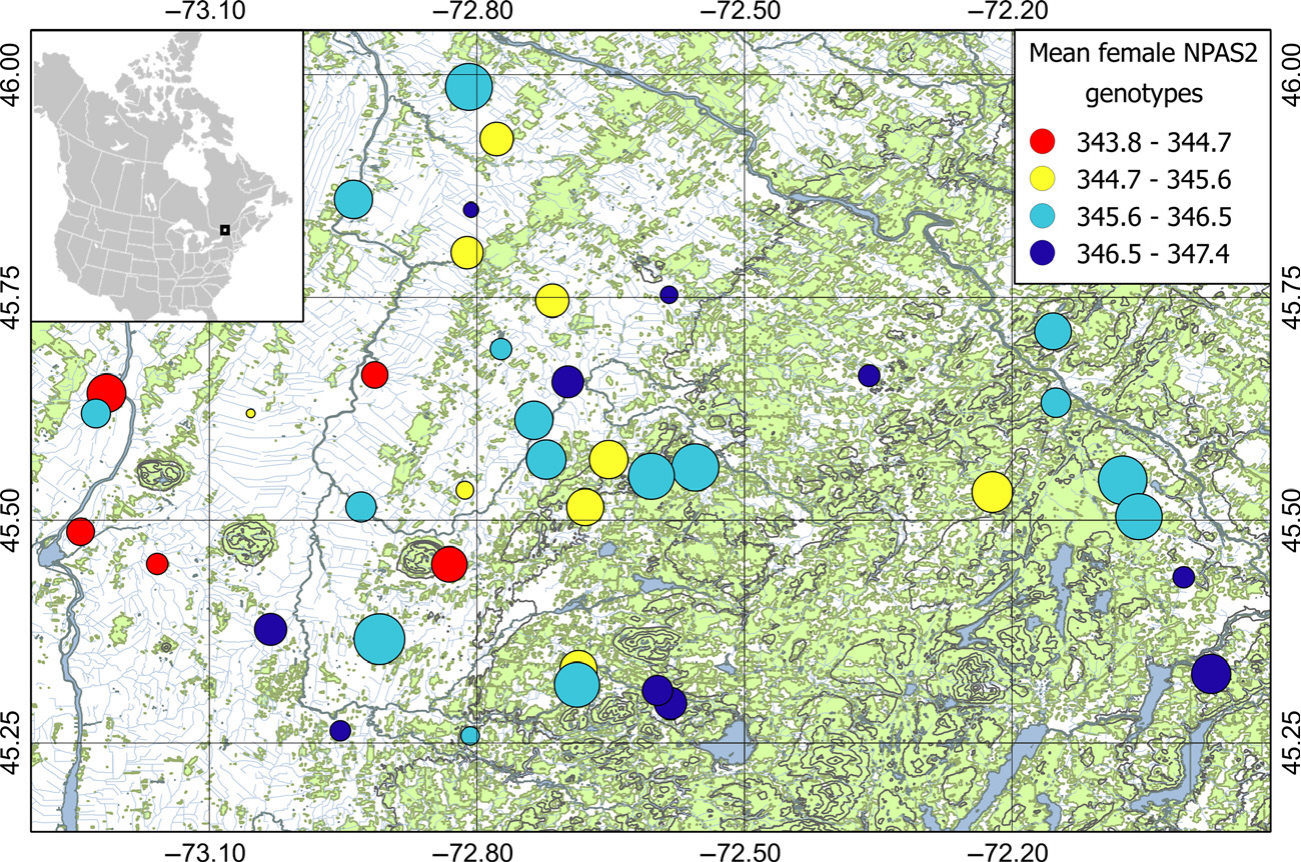
Mean female NPAS2 genotypes observed on the 40 farms (colored circles, see legend) in the study system in southern Québec, Canada. Number of females observed between 2010 and 2013 is represented by different circle sizes (range: 2 – 45). Forest patches (green), rivers and lakes (blue), other land uses (mostly agriculture; white), elevation (100-m gray isolines), latitude and longitude (in decimal degrees; thin black lines) are also represented. This figure was produced with QGIS 2.0 (QGIS Team Development, 2013).

The study system in southern Québec (Canada) covers an area of 10,200 km^2^ and includes 400 nest boxes equally distributed within 40 farms (Fig.1; see Ghilain and Bélisle 2008 for more details on the study system). Between 2010 and 2013, each nest box was visited every 2 days during the reproductive season to record nest box occupation, laying date of the first egg, incubation initiation and hatching date. Incubation duration was calculated as hatching date - incubation initiation date and was highly correlated with the incubation period defined from temperature variation obtained from thermochrons placed within a subset of nest boxes in 2013 (*N* = 34, *r *=* *0.88, *P *<* *0.001; see Appendix S1). Birds were individually identified with an aluminum band (US Fish and Wildlife Service), and females were assigned to an age class, second year (SY), or after second year (ASY), based on feather color (brown or blue-green, respectively; Hussell 1983). DNA was extracted using a salt extraction method from blood samples collected from a brachial vein on a filter paper (Aljanabi and Martinez 1997; Porlier et al. 2009), and its quality and concentration was determined by electrophoreses on 1% agarose gel. The sex of each individual was confirmed with a molecular technique following Lessard et al. (2014). Meteorological data were extracted from 10 meteorological stations located within the study area (Environment Canada, http://meteo.gc.ca/). Time periods showing the strongest correlations with temperature were different for laying date (April 6 – May 9, Bourret et al. unpublished data) and incubation duration (May 18 – June 8, Appendix S1) and are referred hereafter to April and May temperatures, respectively. We only considered first breeding attempts in our analyses, that is, first reproductive event that occurred in a nest box and first record of breeding attempt of both female and social male (if known) within a reproductive season (*N* = 847, see Table S2.1 for details on sample sizes).

### Candidate gene analyses

PCR conditions for CLOCK amplifications were performed following Johnsen et al. (2007) and for ADCYAP1, CREB1, and NPAS2 following Steinmeyer et al. (2009) (details can be found in Table S2.2). We redesigned CREB1 reverse primer (5′-AGAATAACGCAGCCCAGAGC-3′) with Primer-BLAST (Ye et al. 2012) to shorten the PCR product length from ∼550 to ∼280 base pairs and thereby eased PCR amplifications as well as fragments migration and visualization. PCR products were resolved on an AB3130xl automated DNA sequencer, and alleles length were established using GeneMapper 4.1 (Applied Biosystems, Foster City, CA, USA). Between 3 and 11 PCR products of each candidate genes (CLOCK: 3; NPAS2: 4; ADCYAP1: 11; and CREB1: 7) were sent to a sequencing platform (Centre de recherche du CHUL/CHUQ, Québec, Canada; www.sequences.crchul.ulaval.ca) to assess the concordance between targeted genes and PCR products. CLOCK, NPAS2, and CREB1 showed sequences highly similar (>98% identical) to those previously published (Johnsen et al. 2007; Steinmeyer et al. 2009). However, as already reported by Steinmeyer et al. (2009), ADCYAP1 showed an increase of a single base pair between alleles outside of the repeat regions and thus was corrected to reflect the dinucleotide repeat increase. A total of 60 individuals were replicated from DNA extraction to allele scoring (6.5% of all individuals) to assess error rate, which was 1.9% on average for all loci (range: 0.0–3.3%).

Deviations from Hardy–Weinberg equilibrium and heterozygosity were checked for all four candidate genes at different grouping levels (within years, sexes, and age classes) with GenePop 4.0 (Raymond and Rousset 1995; Rousset 2008). Individuals observed more than once were randomly chosen in a single year to avoid pseudo-replication in these analyses. An AMOVA (analysis of molecular variance) was also conducted using Arlequin 3.5 (Excoffier and Lischer 2010) to assess levels of differentiation among years and farms. An individual genotype at a given locus was defined as the sum of allele lengths to represent the additive effect of each allele. This definition of an individual genotype was used because it reflects the suspected effect of STR polymorphism within functional genome regions (Elmore et al. 2012) and it is more powerful statistically than defining distinct factors for each pair of alleles observed (see also Liedvogel et al. 2009 and Mueller et al. 2011 for the rationale behind this method and a comparison between different genotype definitions). Intra-individual correlations between genotypes at each locus, in both males and females, were assessed using Spearman's rank correlation.

### Genetic variation distribution

To assess how genetic variation distribution was related to environmental and spatial variation, we used two approaches. First, we used a linear model implemented in the software R (R Core Team, 2014) to examine the relationship between individual genotypes and environmental components known to influence laying date and/or incubation duration in the study population (laying date: latitude, April temperature and breeding density (A. Bourret, M. Bélisle, F. Pelletier and D. Garant unpublished data); incubation duration: May temperature and longitude (Appendix S1)). To avoid pseudo-replication, individuals were included only once in the analysis and explanatory variables were averaged for individuals observed in more than 1 year (*N* = 220 individuals: 130 females and 90 males). The full model included latitude and longitude (decimal degree), breeding density (% of occupied nest boxes on each farm), temperature (°C), sex and all two-way interactions with sex as explanatory variables. As April and May temperatures were highly correlated within years (*r *>* *0.83), we decided to average these values to a mean temperature. Year was not included in this model because there was no difference in genetic structure among years (see AMOVA results). All explanatory variables were standardized (zero mean, unit variance; Table S1.3) and the final model was determined by sequentially removing the least significant term from the model based on its *P*-value until all remaining variables were significant (*α *=* *0.05) (Crawley 2007). In the second approach, we looked for evidence of spatial autocorrelation in allele-frequency distribution with GenAlEx (Peakall and Smouse 2012). We computed autocorrelation coefficients (*r*) for 10 distance classes of 10 km (covering important distance classes between farms; minimum: 1.9 km, maximum: 103.1 km, and mean (SD): 42.2 (21.1) km) in three datasets: females only, males only, and all individuals. Two-tailed 95% confidence intervals were obtained based on 999 permutations. Individuals observed on more than one farm (*N* = 26, <3% of all individuals) were randomly assigned to a single location.

Genetic variation can also be nonrandomly distributed between mating pairs. To assess the presence of nonrandom mating, we computed the distribution of pairwise genetic relatedness estimator (R_XY_, Lynch and Ritland 1999) between all observed mating pairs (*N* = 485 pairs) for all candidate genes separately (see Mainguy et al. 2009). These distributions were compared with Mann–Whitney *U*-tests to those of all possible male–female pairs within years (*N* = 89,325 pairs), and differences between distributions would suggest nonrandom mating.

### Reproductive parameters and genotypic variations

Laying date, incubation duration, and hatching date are important reproductive parameters potentially correlated with variation in candidate genes (Table1). However, as laying and hatching dates were highly correlated (*r *=* *0.96, *P *<* *0.001), we restricted our analyses to laying date and incubation duration (not correlated, *r *=* *−0.04, *P *=* *0.41). Using linear mixed models, we assessed the relationship between candidate genes and both laying date and incubation duration. As both females and social males showed significant adjusted repeatability for laying date (0.320 and 0.181, respectively) and incubation duration (0.195 and 0.070, respectively; Appendix S3 for more details on repeatability), only clutches with both parents known and genotyped were included in these analyses to disentangle their genotypes relative impact on these traits (Table S2.1 for sample size of each analysis). Full models included as fixed effects: male and female genotypes (continuous), female age class (SY or ASY), relevant environmental variables (same variables as described above plus longitude from the genetic variation distribution analysis, see Results), and all two-way interactions between female age class or environmental covariates and genotypes (except for female age class and CREB1 as some genotype–age class pairs were not observed) to test GxE and genotype–age interactions. Female identity, male identity, and year were included as random effects. Explanatory variables were standardized (zero mean, unit variance), and analyses were performed using lme4 package (Bates et al. 2014) in R. We determined the final models by backward variable selection as explained previously and when a GxE interaction was included in a final model, the main effect of the concerned candidate gene was also assessed from a model without the interaction.

## Results

### Allelic and genotypic variation

We successfully genotyped more than 98.8% of the 925 breeders (554 females, 371 males) captured between 2010 and 2013 (Table2). From the 4 alleles observed at CLOCK, the Q_8_ allele (allele 182) was most frequent (61.6%), a result similar to the observation of Dor et al. (2012) in another tree swallow population. NPAS2 carried 7 different alleles in our study system (most frequent allele: 70.9%), ADCYAP1 was the most polymorphic candidate gene with an observed heterozygosity of 0.825 (13 alleles; most frequent allele: 24.0%) and CREB1 was the least polymorphic candidate gene tested with the most frequent allele accounting for 96.7% of allelic diversity and an observed heterozygosity of 0.064. None of the candidate gene overall allele frequencies deviated from Hardy–Weinberg equilibrium (*P *>* *0.39), neither within years (*P *>* *0.13) nor in female age classes (*P *>* *0.19). However, a closer look within sexes suggested a deviation in males at NPAS2 (*F*_IS_* *=* *−0.019, *P *=* *0.042) that was not significant after Bonferroni correction for multiple comparisons.

**Table 2:** Characteristics of candidate genes analyzed. Sample size genotyped (*N*), number of observed alleles (*N* alleles) and range, number of observed genotypes (*N* genotypes) and range, and observed heterozygosity (*Ho*) for adult tree swallows in this study. A genotype is defined as the sum of observed alleles within an individual

Candidate gene	*N*	*N* alleles	Alleles range	*N* genotypes	Genotypes range	*Ho*
CLOCK	921	4	176–185	6	358–370	0.507
NPAS2	921	7	162–186	9	333–359	0.453
ADCYAP1	914	13	164–188	19	336–372	0.825
CREB1	921	3	261–265	3	524–528	0.064

CLOCK genotypes were weakly correlated with NPAS2 (*r*_*s*_* *=* *0.092, *P *=* *0.041), ADCYAP1 (*r*_*s*_* *=* *−0.114, *P *= 0.011), and CREB1 (*r*_*s*_* *=* *0.099, *P *=* *0.027) in females; however, all results were not significant after Bonferroni corrections. No other correlations among pairs of genotypes for female or for males were significant (all *P *>* *0.21). Finally, in the AMOVA, more than 99% of the total genetic variance was due to individual differences, suggesting no genetic structure among years or farms.

### Environmental effects on genetic variation distribution

The final model suggested an effect of the interaction between sex and longitude on the genotypic distribution of NPAS2 (Sex × Longitude: *β *=* *−0.428 ± 0.203, *t* = 2.11, *P *=* *0.035). A closer examination within each sex revealed a positive significant relationship with longitude in females (*β *=* *0.265 ± 0.124, *t* = 2.13, *P *=* *0.034; Fig.1) but not in males (*β *=* *−0.164 ± 0.163, *t* = 1.00, *P *=* *0.32). No relationships between genotypic and environmental variations were observed for CLOCK, ADCYAP1, and CREB1. Spatial autocorrelation analyses revealed no spatial structure in any of the candidate gene allele distributions (Fig. S4.1). Pairwise genetic relatedness between observed and random mating pairs showed no significant different distributions for all genes (all *P *>* *0.06; Fig. S4.2).

### Genotypic and environmental effects on reproductive parameters

**Figure 2:**
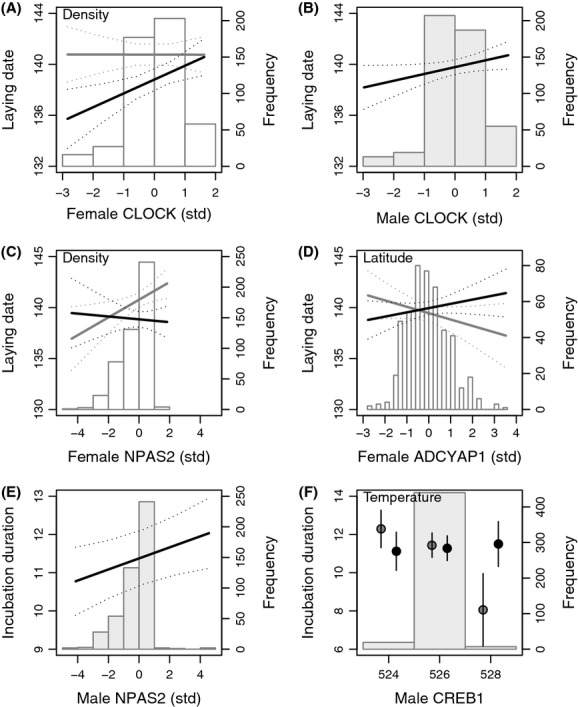
Predictions from the linear mixed models of tree swallow laying date (Julian days; A-D) and incubation duration (days; E-F) correlate with candidate gene genotypes (CLOCK: A, B; NPAS2: C, E; ADCYAP1: D; CREB1: F). Interactions with breeding density (A, C), latitude (D) and May temperature (F) are presented for the first (gray) and third (black) quartile of environmental values. Genotype frequency histograms for male (gray) or female (white) and 95% confidence intervals of predictions (from models with year included as a fixed effect) are also presented on each panel.

Laying date was a function of the polymorphism at three candidate genes (Fig.2A–D, Table3). First, laying date showed a positive relationship with CLOCK female genotypes in interaction with breeding density – with a steeper slope at higher density (Fig.2A) – and a positive relationship with CLOCK male genotypes, albeit marginally nonsignificant (*P *=* *0.084, Fig.2B). As for CLOCK model, an interaction between NPAS2 female genotypes and breeding density was kept in the final NPAS2 model (Fig.2C). However, the relationship with laying date in this case seemed null at higher density but turned positive at lower density. Finally, ADCYAP1 female genotypes also showed a relationship with laying date, but this time in interaction with latitude – with a negative slope at lower latitude turning positive at higher latitude (Fig.2D). None of the variables were kept in the final CREB1 model (Table S4.4). Main effects of CLOCK (*β *=* *0.510 ± 0.294, *t* = 1.74, *P *=* *0.08), NPAS2 (*β *=* *0.355 ± 0.291, *t* = 1.22, *P *=* *0.22), and ADCYAP1 (*β *=* *0.106 ± 0.292, *t* = 0.36, *P *=* *0.72) female genotypes were all nonsignificant.

**Table 3:** Final linear mixed models analyses of laying dates for (a) CLOCK, (b) NPAS2, and (c) ADCYAP1 male and female genotypes. Female age class (SY or ASY) and environmental variables were included as fixed effects and tested for interactions with breeder genotypes. Year, female identity, and male identity were included as random effects and all explanatory variables were standardized. None of the variables were kept in the final CREB1 model. Full models can be found in Appendix S4

Models	Variables	Estimates	SE	*t*-value	*P*-value
(a) CLOCK	Intercept	138.919	0.877	158.39	<0.001
	Density	−1.252	0.297	4.22	<0.001
	CLOCK male	0.493	0.287	1.72	0.087
	CLOCK female	0.556	0.296	1.88	0.061
	CLOCK female × Density	0.659	0.295	2.24	0.026
(b) NPAS2	Intercept	138.957	1.216	114.29	<0.001
	Density	−1.136	0.295	3.86	<0.001
	NPAS2 female	0.309	0.290	1.07	0.29
	NPAS2 female × Density	−0.700	0.280	2.50	0.013
(c) ADCYAP1	Intercept	138.935	0.873	159.17	<0.001
	Latitude	0.372	0.306	1.22	0.23
	ADCYAP1 female	0.103	0.293	0.35	0.73
	ADCYAP1 female × Latitude	0.697	0.316	2.21	0.028

Incubation duration varied as a function of male genotypes at two candidate genes (Fig.2E,F; Table4). NPAS2 male genotypes showed a positive relationship with incubation duration, with 1.2 day difference for most distant genotypes (Fig.2E). CREB1 male genotypes in interaction with May temperature showed a negative relationship with incubation duration (Table4; this last model was refitted with CREB1 genotypes defined as factorial variables and a similar interaction was observed, Fig.2F), but the main effect was not significant after removing the GxE interaction (*β *=* *−0.076 ± 0.621, *t* = 1.22, *P *= 0.22). No significant relationships between incubation duration and genotypes were found for CLOCK and ADCYAP1 models, although a marginally nonsignificant effect was observed for the interaction between CLOCK male genotype and both longitude (*P *=* *0.06) and May temperature (*P *=* *0.08) (Table S4.5). Full models for laying date and incubation duration are detailed in Appendix S4.

**Table 4:** Final linear mixed models analyses of incubation duration for a) NPAS2 and b) CREB1 male and female genotypes. Female age class (SY or ASY) and environmental variables were included as fixed effects and tested for interactions with breeder genotypes. Year, female identity, and male identity were included as random effects, and all explanatory variables were standardized. None of the variables were kept in the final CLOCK and ADCYAP1 models. Full models can be found in Appendix S4

Models	Variables	Estimates	SE	*t*-value	*P*-value
(a) NPAS2	Intercept	11.323	0.360	31.43	<0.001
	NPAS2 male	0.144	0.063	2.28	0.023
(b) CREB1	Intercept	11.301	0.313	36.14	<0.001
	Temperature	−0.101	0.094	1.07	0.29
	CREB1 male	−0.109	0.062	1.76	0.079
	CREB1 male × Temperature	0.218	0.058	3.75	<0.001

## Discussion

We investigated the relationship between four candidate genes (CLOCK, NPAS2, ADCYAP1, and CREB1) and two phenological traits related to reproduction: laying date and incubation duration. We used 4 years of data for males and females and included in our statistical analysis gene–environment interactions (GxE) to account for potential confounding environmental effects. We observed relationships between length polymorphism at all candidate genes and laying date and/or incubation duration. Most of these relationships were affected by environmental variables (breeding density, latitude, or temperature), emphasizing the presence and importance of GxE in our study system and its potential role in explaining divergent results among previous studies (see references in Table1).

### Polymorphism and spatial variation at candidate genes

Number of alleles and heterozygosity observed for CLOCK, NPAS2, and ADCYAP1 genes in this study were similar to those reported in other bird species (see Appendix S5 for a review of allelic diversity reported in other studies). Notably, CLOCK allele frequencies previously reported in a tree swallow population in Ithaca (NY, USA) by Dor et al. (2012) were almost identical to those observed in our study. In contrast, CREB1 number of alleles and heterozygosity were lower in our study system (*N* = 3 alleles, *Ho *=* *0.064) than in most previous reports from other species (Table S5.1, *N* = 6−10 alleles, *Ho *=* *0.267−0.300), the only exception being the study by Chakarov et al. (2013) on raptors (*N* = 1−3 alleles, *Ho *=* *0.093).

The presence of genetic variation in relation to space and/or environmental components can indicate underlying evolutionary processes, but also functional roles when observed at candidate genes (Fitzpatrick et al. 2005). For example, latitudinal clines observed in CLOCK allele lengths suggested local adaptations to the photoperiodic gradient in some species and thus a functional role for its length polymorphism (e.g., Johnsen et al. 2007; O'Malley and Banks 2008; reviewed in Kyriacou et al. 2008). Here, we did not observe a latitudinal cline in CLOCK genotypes within our study system. Also, the similarity of our population with the tree swallow population in Ithaca in terms of CLOCK allele frequencies despite a latitudinal distance of approx. 3° suggests an absence of latitudinal cline at larger spatial scale for this species. However, the longitudinal cline observed for NPAS2 female genotypes could be in part linked to the genetic basis of timing of migration. A previous study using microsatellites in the same system found no strong genetic structure in space, but still a tendency for more genetically similar individuals to be more geographically distant, an observation that is contrary to any spatial cline or isolation by distance patterns (Porlier et al. 2009). The pattern of alleles distribution observed at NPAS2 was thus different from the pattern observed at putatively selectively neutral microsatellite loci, which suggests an adaptive role to the observed NPAS2 cline. Furthermore, the same study by Porlier et al. (2009) showed that settlement dates in nest boxes were positively correlated with farm distance to the St. Lawrence River, itself highly correlated with longitude (*r *=* *0.90), revealing a possible migration route from west to east within the study area. Taken together with our results, these observations suggest that earlier settlement dates could be related to shorter NPAS2 female genotypes.

### Laying date versus candidate genes

We found a positive relationship between CLOCK female genotypes and laying date, which supports the results previously reported in blue tits and barn swallows of earlier laying dates at smaller allele length (Liedvogel et al. 2009; Caprioli et al. 2012). However, in our case, we found evidences of GxE as this relationship was influenced by breeding density with a steeper slope at higher densities. Our result also contrasts with those obtained in the Ithaca tree swallow population by Dor et al. (2012) where the relationship between CLOCK female genotypes and laying date was nonsignificant (*β *=* *0.494 ± 0.612, *F*_1,462_ = 0.65, *P *=* *0.42, Dor et al. 2012) despite similar sample size. In that previous study, however, GxE was not considered which may explain the discrepancy. In fact, when applying the statistical model used by Dor et al. (2012) (i.e., linear mixed model with age and year as fixed effects, female identity as random effect and female genotype defined as CLOCK poly-Q average allele size) to our dataset, we also found a nonsignificant relationship between CLOCK female genotypes and laying date (*β *=* *1.033 ± 0.672, *t* = 1.54, *P *=* *0.13).

The relationships between timing of reproduction and NPAS2, ADCYAP1, or CREB1 were previously tested only once in birds, in a study on the common buzzard that found no significant associations (Chakarov et al. 2013; Table1). To our knowledge, we thus provide the first evidence of relationships between variation at NPAS2 and ADCYAP1 and laying date in birds. Similarly to the results obtained for CLOCK, the relationship between NPAS2 female genotypes and laying date was affected by breeding density, but in a different fashion. In fact, while for CLOCK the laying date–genotype relationship was steeper at higher breeding density, for NPAS2, the relationship was steeper at lower breeding density. Despite the fact that CLOCK and NPAS2 are paralogs and have partially overlapping functions within the circadian system (Debruyne 2008), they may not be affected in the same manner by a given environmental variable – which emphasizes the importance of considering several genes when assessing GxE.

We also found a correlation between ADCYAP1 female genotypes and laying date in interaction with latitude. In previous studies, ADCYAP1 longer genotypes were associated to greater migratory restlessness (blackcaps (*Sylvia atricapilla*), Mueller et al. 2011; Oregon juncos (*Junco hyemalis thurberri*), Peterson et al. 2013) and to a tendency to disperse earlier in the season (common buzzards, Chakarov et al. 2013). In line with the NPAS2 cline reported here, we could speculate that the latitudinal difference found for the laying date–ADYCAP1 genotype relationship in females is linked to spatial variation in migratory patterns, but this should be further investigated. Again, for both NPAS2 and ADCYAP1, conducting analyses without including GxE effects would have resulted in nonsignificant relationships, emphasizing the importance of environmental interactions in the relationships observed here.

Finally, it is worth noting that the relationship observed between laying date and CLOCK male genotypes, despite being marginally nonsignificant, was similar in direction and effect size to the equivalent relationship in females. This result is concordant with the small repeatability observed for this trait in males (Appendix S3). The male component of genetic variation was rarely taken into account in previous studies of candidate gene variation effects on timing of reproduction (i.e., Table1). This is somewhat surprising given the moderate male repeatability and/or heritability documented for laying date in some bird species (e.g., mute swans (*Cygnus olor*), Charmantier et al. 2006 and Auld et al. 2013; common gulls (*Larus canus*), Brommer and Rattiste 2008; tawny owls (*Strix aluco*), Brommer et al. 2015). In red-billed gulls (*Larus novaehollandiae*), for example, significant additive genetic variance component and nonzero heritability for laying date were reported in males (Teplitsky et al. 2010). The authors suggested that this effect was due to the male influence on its partner through courtship feeding behavior prior to the laying of the eggs. Here, the male CLOCK length polymorphism influence on laying date could be explained, for example, by individual differences in arrival date or by differences in capacity to select optimal site for breeding, which could in turn affect their partner timing of breeding.

### Incubation duration versus candidate genes

Our knowledge of the genetic architecture underlying incubation duration in birds is minimal. Few studies have investigated the relationship between this trait and polymorphism at candidate genes (and for CLOCK only, see Table1) despite limited but nonzero heritability in one out the three species studied so far (collared flycatchers (*Ficedula albicollis*): female *h*^2^* *=* *0.040, Husby et al. 2012; but see Liedvogel et al. 2012 for zero heritability in great tits (*Parus major*) and blue tits). Repeatability, the superior limit of heritability (Boake 1989; Falconer and Mackay 1996; but see Dohm 2002 for some limitations) was significantly different from zero here in both sexes (females: 0.195; males: 0.070; see Appendix S3), suggesting potential for nonzero heritability in incubation duration, although a detailed quantitative genetics analysis will be required to verify this assessment. In our analyses, male NPAS2 and CREB1 genotypes were correlated with incubation duration. These results were somewhat unexpected given that the role of male tree swallows during incubation is hypothesized to be negligible – they do not incubate, nor do they feed their mates during this period (Winkler et al. 2011). However, males participate in nest building and line their nest with feathers, which could indirectly influence incubation duration (Lombardo et al. 1995). Circadian components such as NPAS2 and CREB1 could also be related to male behavior and indirectly influence female behavior. For example, in great tits, a trait related to the circadian rhythm, the free-running period length, was suspected to be associated with male reproductive behavior. This highly heritable trait (*h*^2^* *=* *0.86) showed smaller value in extra-pair young than within-pair young (Helm and Visser 2010), suggesting that extra-pair males had shorter free-running period length than the males they cuckolded. Behavioral influence related to candidate genes could possibly be indirectly acting here and this certainly deserves further investigation.

### Importance of GxE

Despite the importance of genotype–environment interactions in evolutionary biology (Via and Lande 1985; Fitzpatrick et al. 2005; Saastamoinen et al. 2009; Bourret et al. 2013), GxE involving candidate genes have been little studied in the wild in relation to phenological traits related to reproduction (see Table1). In previous avian studies, GxE interactions were only tested for CLOCK in blue tits (Liedvogel et al. 2009) and great tits (Liedvogel and Sheldon 2010) of Wytham Woods, UK. In these studies, all ecological variables known to influence reproduction timing for these populations (i.e., altitude, oak richness, and breeding density) were included, but no GxE was detected. The GxE interactions reported at all candidate genes considered in our study suggest that their importance may vary depending on the environmental context and/or species. For example, migratory species, including Tree swallow, are likely to be subjected to selective pressures on their circadian system that are distinct from those affecting resident species such as the Blue tit and Great tit of Wytham Woods (Liedvogel et al. 2011). Flexibility to adjust to local conditions could evolve through GxE in migratory species and this could explain the discrepancy between previous studies and this one.

Importantly, some precautions are needed when interpreting GxE interactions because of potential publication bias favoring significant results, high false-positive rate when assessing multiple comparisons and rare replications of previous findings (Little et al. 2009; Duncan and Keller 2011). The multiple comparisons problem is particularly important in studies assessing GxE interactions using numerous SNPs because of the massive increasing of type-2 errors that need to be accounted for, but this problem can still hold in this study at a smaller scale because we used four different candidate genes. However, applying a strict Bonferroni correction to our analyses (i.e., reduce the alpha level for a significant *P*-value to 0.013) still suggests the importance of one candidate gene–environmental interaction for laying date and incubation duration (NPAS2 female genotype−breeding density and CREB1 male genotype−May temperature interactions, respectively). Nonetheless, we believe that replicates should be obtained from other tree swallow populations and more species for a better understanding of the importance of GxE involving these phenological candidate genes.

Finally, the detection of relevant GxE interactions is also dependent on the underlying neutral population genetic structure and the validity and reliability of the environmental variables used when testing for such effects. Here, a previous study on this tree swallow population showed no important genetic structure at neutral microsatellite loci (Porlier et al. 2009; see Polymorphism and spatial variation at candidate genes discussion above), suggesting that observed patterns at candidate genes were not due to spurious associations caused by population structure. Moreover, we only assessed the effect of environment variables known to have a direct influence on the trait of interest or on the genotype spatial distribution to reduce potential bias in our candidate gene analyses (see Little et al. 2009; Saïdou et al. 2014). Indeed, the environmental variables (i.e., breeding density, latitude, and spring temperature) interacting with at least one candidate gene in this study were all previously known to influence bird phenology. First, breeding density was identified as the main determinant of laying date in this population and is hypothesized to be a good proxy of environmental quality (A. Bourret, M. Bélisle, F. Pelletier and D. Garant unpublished data). In fact, differences in habitat quality are thought to lead to aggregation of tree swallows in habitats with more food (Dunn and Winkler 1999), a situation that can be transposed in our nest box system given both the preference of tree swallows for nest boxes over natural cavities and the presence of empty nest boxes in most environments (reviewed in Shutler et al. 2012). As previously reported, lower breeding density environments were associated with later laying dates (see Dunn and Winkler 1999), but our results also suggest that the effect of this environmental constraint varied depending on female CLOCK and NPAS2 genotypes. The second environmental variable, latitude, is tightly linked to photoperiod (Dawson 2013) and can influence laying date even at a small spatial scale (Gienapp et al. 2010). However, the antagonistic interaction observed here cannot be easily interpreted given the current sparse knowledge of ADCYAP1 influence on phenological traits in other tree swallow populations and other species. The last interacting environmental variable, spring temperature, influences incubation duration and partly controls male gonad maturation (Dawson 2008). The GxE interaction observed at CREB1 in males seems due to the presence of the genotype 528, which showed variable incubation duration depending on temperature. This result is even more interesting given the low frequency of the genotype 528 in our population (i.e., 2.1%), which suggests that underlying evolutionary processes such as selection could have affected this particular genotype.

## Conclusion

Candidate gene approaches provide complementary information to quantitative genetic studies (Liedvogel et al. 2012) and offer a direct window on the potential for evolutionary changes. Most phenotypic traits are hypothesized to be under the control of numerous genes with small effect sizes (Manolio et al. 2009). As a result, the power to detect relationships between phenotypes and variation at candidate genes rely on gene effect sizes, heritabilities, allele frequencies and sample sizes (Manolio et al. 2009; Liedvogel et al. 2012; Saïdou et al. 2014). In the wild, variable environmental conditions and interacting ecological and genetic components complicate candidate gene studies. Despite all these constraints, we managed to document relationships between variation at four candidate genes and phenological traits and provided evidences of GxE. Altogether, our results suggest that CLOCK, NPAS2, ADCYAP1, and CREB1 can be good candidate genes to monitor and to predict future adaptation to changing environmental conditions if the environmental context in which they are expressed is taken into account.

## Data accessibility

Data available from the Dryad Digital Repository: http://dx.doi.org/10.5061/dryad.f7t25.

## Supporting Information

Appendix S1. Environmental determinants of incubation duration. **Table S1.** Final linear model of the environmental determinants of mean incubation duration (*N* = 148) in tree swallows. **Appendix S2.** Supplementary information on methods. **Table S2.1.** Sample size of breeders (males and females) and nests (for first breeding attempts only) used in this study for laying date/incubation duration of tree swallows breeding between 2010 and 2013 in the southern Québec study system. **Table S2.2.** Details of PCR products and amplification conditions for candidate gene amplifications, following Jonhsen et al. (2008; CLOCK) and Steinmeyer et al. (2009; ADCYAP1, CREB1, NPAS2). **Table S2.3.** Descriptive statistics of variables included in the statistical analyses prior to standardization (zero mean and unit variance), for A) the environmental effects on genetic variation distribution analysis (see Table2) and B) the genotypic effects on reproductive parameters analysis (see Table3). **Appendix S3.** Adjusted repeatability for laying date and incubation duration. **Appendix S4.** Supplementary results. **Figure S4.1.** Spatial autocorrelation analysis for A) CLOCK, B) NPAS2, C) ADCYAP1 and D) CREB1 alleles of tree swallows breeding between 2010 and 2013 in this study. **Figure S4.2.** Distribution of estimates of pairwise genetic relatedness (*r*) of observed mating pairs (white bar) and random mating pairs (black bar) of tree swallows breeding between 2010 and 2013 in this study for A) CLOCK, B) NPAS2, C) ADCYAP1 and D) CREB1. **Table S4.1.** Full linear mixed model of laying date for CLOCK male and female genotypes. **Table S4.2.** Full linear mixed model of laying date for NPAS2 male and female genotypes. **Table S4.3.** Full linear mixed model of laying date for ADCYAP1 male and female genotypes. **Table S4.4.** Full linear mixed model of laying date for CREB1 male and female genotypes. **Table S4.5.** Full linear mixed model of incubation duration for CLOCK male and female genotypes. **Table S4.6.** Full linear mixed models of incubation duration for NPAS1 male and female genotypes. **Table S4.7.** Full linear mixed model of incubation duration for ADCYAP1 male and female genotypes. **Table S4.8.** Full linear mixed model of incubation duration for CREB1 male and female genotypes. **Appendix S5.** Supplementary information on allelic diversity. **Table S5.1.** Summary of studies assessing allelic diversity for CLOCK, NPAS2, ADCYAP1 and CREB1 in various bird species. Number of observed alleles (*N* alleles) and observed heterozygosity (*Ho*) are reported. **Appendix S6.** References.
