# Supplementary material for: Candidate gene–environment interactions and their relationships with timing of breeding in a wild bird population
Source: Ecol Evol. 2015 Aug 11;5(17):3628–41. doi: 10.1002/ece3.1630 (PMC4567867; doi:10.1002/ece3.1630)
Supplement: Appendix S1. — Environmental determinants of incubation duration. Table S1. Final linear model of the environmental determinants of mean incubation duration (N = 148) in tree swallows. Appendix S2. Supplementary information on methods. Table S2.1. Sample size of breeders (males and females) and nests (for first breeding attempts only) used in this study for laying date/incubation duration of tree swallows breeding between 2010 and 2013 in the southern Québec study system. Table S2.2. Details of PCR products and amplification conditions for candidate gene amplifications, following Jonhsen et al. (2008; CLOCK) and Steinmeyer et al. (2009; ADCYAP1, CREB1, NPAS2). Table S2.3. Descriptive statistics of variables included in the statistical analyses prior to standardization (zero mean and unit variance), for A) the environmental effects on genetic variation distribution analysis (see Table2) and B) the genotypic effects on reproductive parameters analysis (see Table3). Appendix S3. Adjusted repeatability for laying date and incubation duration. Appendix S4. Supplementary results. Figure S4.1. Spatial autocorrelation analysis for A) CLOCK, B) NPAS2, C) ADCYAP1 and D) CREB1 alleles of tree swallows breeding between 2010 and 2013 in this study. Figure S4.2. Distribution of estimates of pairwise genetic relatedness (r) of observed mating pairs (white bar) and random mating pairs (black bar) of tree swallows breeding between 2010 and 2013 in this study for A) CLOCK, B) NPAS2, C) ADCYAP1 and D) CREB1. Table S4.1. Full linear mixed model of laying date for CLOCK male and female genotypes. Table S4.2. Full linear mixed model of laying date for NPAS2 male and female genotypes. Table S4.3. Full linear mixed model of laying date for ADCYAP1 male and female genotypes. Table S4.4. Full linear mixed model of laying date for CREB1 male and female genotypes. Table S4.5. Full linear mixed model of incubation duration for CLOCK male and female genotypes. Table S4.6. Full linear mixed models of incubation [file ece30005-3628-sd1.docx]

**Supporting Information**

candidate gene-environment interactions and their relationships with timing of breeding in a wild bird population

Audrey Bourret and Dany Garant

***S1.*** *Environmental determinants of incubation duration*

The method used to define incubation duration in this study (i.e. [hatching date - incubation initiation date]) differed slightly from the traditional way of calculating incubation duration (i.e. hatching date – [laying date + clutch size – 1]). In fact, the method used here gives a better fit with incubation duration obtained from thermocrons placed within a subset of nest boxes in 2013 (N=34, *r*=0.88, *P*<0.001) compared to the traditional method (N=34, *r*=0.19, *P*=0.27). These thermocrons collected temperature every 2 minutes from a few days after laying date till hatchling date and days with abrupt temperature increase were considered as incubation initiation date.

For incubation analysis, daily mean temperature (°C) and daily rainfall (mm) were obtained from sliding windows analyses following the methodology described in Bourret *et al.* (submitted) with slight modifications. We tested windows varying from 5 to 121 days, from Julian days 60 to 181 (respectively March 1 and June 31 in non-leap year) for a total of 6903 windows. The strongest correlation of incubation duration and mean temperature was between Julian day 138 and 159 (May 18 – June 8; *r*=-0.999, *P*<0.001, while for rainfall, this window was between Julian day 105 and 117 (April 15 – 27; *r*=-0.990, *P*=0.010). We then used these periods as our references for computing both annual mean temperatures and annual rainfalls of ten meteorological stations near our farms.

We investigated the relative importance of different environmental variables on annual mean incubation duration observed on a farm (N=148, 12 farm-year with no observations) with a linear model (farm identity were tested as random effect using a Likelihood Ratio Test (LRTs) but was not significant and thus we used a simple linear model). The full model included annual mean temperature and rainfall (from sliding window analysis), breeding density (% of nest boxes occupied on a farm), longitude and latitude (decimal degree) and two-way interactions with breeding density. We did not include year and elevation as they were both highly correlated with temperature (*r*=0.74) and elevation (*r*=0.92), respectively. All explanatory variables were standardized (zero mean, unit variance, Table S2.3). We determined the final model by sequentially removing the least significant term from the model based on its *P*-value until all remaining variables were significant (α=0.05) (Crawley 2007). This model suggest a negative relationship between incubation duration and both longitude and temperature (Table S1).

**Table S1** – Final linear model of the environmental determinants of mean incubation duration (N=148) in tree swallows. Explanatory variables were standardized and adjusted *R*^2^ for fixed effects was 0.195.

| Variables | Estimates | S.E. | t-value | *P*-value |
| --- | --- | --- | --- | --- |
| Intercept | 11.340 | 0.076 | 149.87 | <0.001 |
| Longitude | -0.233 | 0.084 | 2.79 | 0.006 |
| Temperature | -0.513 | 0.084 | 6.14 | <0.001 |

***S2.*** *Supplementary information on methods*

**Table S2.1** – Sample size of breeders (males and females) and nests (for first breeding attempts only) used in this study for laying date/incubation duration of tree swallows breeding between 2010 and 2013 in the southern Quebec study system.

|  | 2010 | 2011 | 2012 | 2013 | Total |
| --- | --- | --- | --- | --- | --- |
| N clutches | 217/165 | 214/157 | 200/154 | 216/124 | 847/600 |
| with 2 adults known | 142/133 | 121/118 | 119/117 | 103/101 | 485/469 |
| with 2 adults known and genotyped | 142/133 | 119/116 | 119/117 | 102/100 | 470/466 |

**Table S2.2** – Details of PCR products and amplification conditions for candidate gene amplifications, following Jonhsen *et al.* (2008; CLOCK) and Steinmeyer *et al.* (2009; ADCYAP1, CREB1, NPAS2).

|  | CLOCK | NPAS2 | ADCYAP1 | CREB1 |
| --- | --- | --- | --- | --- |
| PCR products | | | | |
| Buffer GOLD (1X) | 1 | 1 | 1 | 1 |
| MgCl_2_ (mM) | 2.5 | 2 | 1 | 2 |
| dNTPs (µM) | 0.20 | 0.20 | 0.20 | 0.20 |
| BSA (mg/ml) | 0.40 | - | - | - |
| Reverse primer (mM) | 0.50 | 0.50 | 0.50 | 0.50 |
| Forward primer (mM) | 0.25 | 0.50 | 0.50 | 0.50 |
| Taq – Amplitaq Gold – Life technologies (U) | 1 | 1 | 1 | 1 |
| DNA (ng) | 10 | 10 | 10 | 10 |
| PCR amplification conditions | | | | |
| Initial denaturation | 92 °C / 3 min | 95 °C / 3 min | 95 °C / 3 min | 95 °C / 3 min |
| Denaturation | 92 °C / 30 s | 95 °C / 30 s | 95 °C / 30 s | 95 °C / 30 s |
| Annealing | 53 °C / 30 s | 53 °C / 30 s | 51 °C / 30 s | 55 °C / 30 s |
| Elongation | 72 °C / 30 s | 72 °C / 60 s | 72 °C / 60 s | 72 °C / 60 s |
| Final elongation | 72 °C / 30 s | 72 °C / 7 min | 72 °C / 7 min | 72 °C / 7 min |
| N cycles | 35 | 35 | 35 | 35 |

**Table S2.3** – Descriptive statistics of variables included in the statistical analyses prior to standardization (zero mean and unit variance), for A) the environmental effects on genetic variation distribution analysis (see Table 2) and B) the genotypic effects on reproductive parameters analysis (see Table 3).

| Analysis | Environmental  Variable | Range | Mean | Standard deviation |
| --- | --- | --- | --- | --- |
| A) Environmental effects on genetic variation distribution | Breeding density | 0.1 – 1.0 | 0.7 | 0.2 |
|  | Latitude | 45.26 – 45.99 | 45.56 | 0.18 |
|  | Longitude | -73.24 – -71.98 | -72.65 | 0.33 |
|  | Mean temperature | 10.95 – 14.19 | 12.52 | 0.75 |
| B) Genotypic effects on reproductive parameters | Breeding density | 0.1 – 1.0 | 0.7 | 0.2 |
|  | Latitude | 45.26 – 45.99 | 45.55 | 0.18 |
|  | Longitude | -73.24 – -71.98 | -72.64 | 0.33 |
|  | April temperature | 6.21 – 10.57 | 8.38 | 1.11 |
|  | May temperature | 13.75 – 18.86 | 16.69 | 1.15 |
|  | CLOCK female | 358 – 370 | 365.6 | 2.7 |
|  | CLOCK male | 358 – 370 | 365.6 | 2.6 |
|  | NPAS2 female | 333 – 351 | 345.7 | 2.9 |
|  | NPAS2 male | 333 – 360 | 345.8 | 3.1 |
|  | ADCYAP1 female | 336 – 368 | 350.1 | 5.1 |
|  | ADCYAP1 male | 338 – 370 | 350.4 | 5.3 |
|  | CREB1 female | 524 – 528 | 526.0 | 0.6 |
|  | CREB1 male | 524 – 528 | 525.9 | 0.5 |

***S3.*** *Adjusted repeatability for laying date and incubation duration*

We computed adjusted repeatability (R_adj_) for laying date and incubation duration to assess the individual identity influence on these traits. To disentangle the relative impact of female and social male identity, we calculated R_adj_ from a linear mixed model where both identities were included as random effects (Liedvogel *et al.* 2009; Chakarov *et al.* 2013). We also included in this model female age class and relevant environmental variables described in the main text as fixed effects and year as random effect to account for possible confounding factors (year could not be included as a fixed effect because it was highly correlated with May temperature; *r*=-0.72, *P*<0.001, VIF=3.53) (Nakagawa & Schielzeth 2010).

For laying date, the adjusted repeatabilities for females and males were 0.320 and 0.181, respectively, while for incubation duration, it was 0.195 for females and 0.070 for male.

***S4.*** *Supplementary results*

**Figure S4.1** – Spatial autocorrelation analysis for A) CLOCK, B) NPAS2, C) ADCYAP1 and D) CREB1 alleles of tree swallows breeding between 2010 and 2013 in this study. Females (blue), males (red) and all individuals (black) were tested for 10 distance classes (circle). 95% confidence intervals are presented for both sexes and overall (dotted lines).


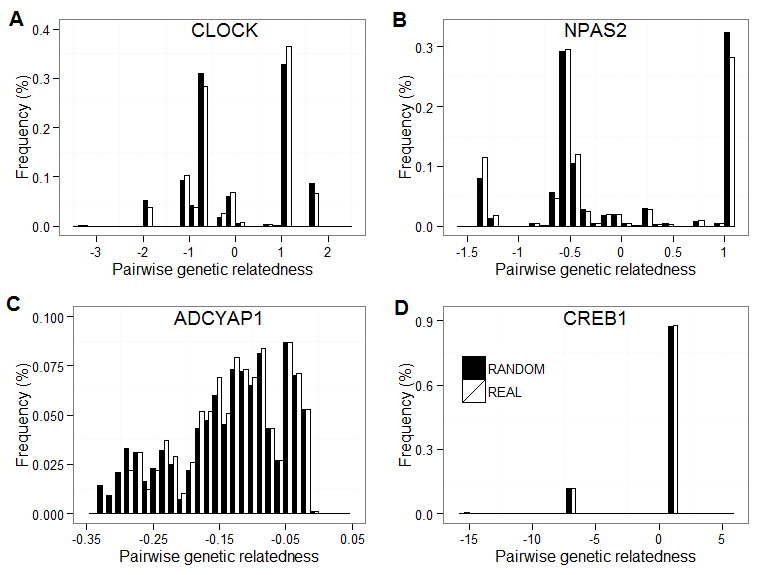


**Figure S4.2** – Distribution of estimates of pairwise genetic relatedness (*r*) of observed mating pairs (white bar) and random mating pairs (black bar) of tree swallows breeding between 2010 and 2013 in this study for A) CLOCK, B) NPAS2, C) ADCYAP1 and D) CREB1.

**Table S4.1** – Full linear mixed model of laying date for CLOCK male and female genotypes. Female age class and environmental variables were included as fixed effects and tested for interactions with breeder genotypes. Female identity, male identity and year were included as random effects and all explanatory variables were standardized. Bold variables were kept in the final model.

| Variables | Estimates | S.E. | t-value | *P*-value |
| --- | --- | --- | --- | --- |
| Intercept | **138.919** | **0.877** | **158.39** | **<0.001** |
| Age | **5.440** | **0.705** | **7.72** | **<0.001** |
| Density | **-1.252** | **0.297** | **4.22** | **<0.001** |
| April temperature | **-1.058** | **0.479** | **2.21** | **0.033** |
| CLOCK female | **0.556** | **0.296** | **1.88** | **0.061** |
| CLOCK female X Density | **0.659** | **0.295** | **2.24** | **0.026** |
| CLOCK male | 0.496 | 0.286 | 1.73 | 0.084 |
| Latitude | 0.294 | 0.303 | 0.97 | 0.33 |
| Longitude | -0.121 | 0.395 | 0.31 | 0.76 |
| CLOCK male X April temperature | 0.326 | 0.274 | 1.19 | 0.23 |
| CLOCK female X Age | -0.719 | 0.702 | 1.02 | 0.31 |
| CLOCK female X Latitude | -0.105 | 0.320 | 0.33 | 0.74 |
| CLOCK male X Longitude | -0.360 | 0.331 | 1.09 | 0.28 |
| CLOCK male X Density | -0.264 | 0.293 | 0.90 | 0.37 |
| CLOCK female X April temperature | -0.239 | 0.269 | 0.89 | 0.38 |
| CLOCK male X Longitude | 0.212 | 0.361 | 0.59 | 0.55 |
| CLOCK female X Latitude | -0.123 | 0.319 | 0.39 | 0.70 |
| CLOCK female X CLOCK male | -0.072 | 0.256 | 0.28 | 0.78 |
| CLOCK male X Age | 0.143 | 0.612 | 0.23 | 0.82 |

**Table S4.2** – Full linear mixed model of laying date for NPAS2 male and female genotypes. Female age class and environmental variables were included as fixed effects and tested for interactions with breeder genotypes. Female identity, male identity and year were included as random effects and all explanatory variables were standardized. Bold variables were kept in the final model.

| Variables | Estimates | S.E. | t-value | *P*-value |
| --- | --- | --- | --- | --- |
| Intercept | **138.957** | **1.216** | **114.29** | **<0.001** |
| Age | **5.327** | **0.707** | **7.53** | **<0.001** |
| Density | **-1.136** | **0.295** | **3.86** | **<0.001** |
| NPAS2 female | **0.309** | **0.290** | **1.07** | **0.29** |
| NPAS2 female X Density | **-0.700** | **0.280** | **2.50** | **0.013** |
| April temperature | -0.917 | 0.482 | 1.90 | 0.064 |
| NPAS2 male | -0.405 | 0.284 | 1.43 | 0.15 |
| Latitude | 0.331 | 0.303 | 1.09 | 0.28 |
| Longitude | -0.057 | 0.397 | 0.14 | 0.89 |
| NPAS2 male X Density | -0.355 | 0.288 | 1.23 | 0.22 |
| NPAS2 female X NPAS2 male | -0.353 | 0.316 | 1.12 | 0.27 |
| NPAS2 female X April temperature | 0.296 | 0.266 | 1.11 | 0.27 |
| NPAS2 female X Age | 0.731 | 0.704 | 1.04 | 0.30 |
| NPAS2 male X Longitude | 0.261 | 0.337 | 0.78 | 0.44 |
| NPAS2 male X April temperature | 0.282 | 0.296 | 0.96 | 0.34 |
| NPAS2 male X Age | 0.607 | 0.714 | 0.85 | 0.40 |
| NPAS2 female X Latitude | 0.119 | 0.318 | 0.37 | 0.71 |
| NPAS2 male X Latitude | 0.065 | 0.371 | 0.23 | 0.82 |
| NPAS2 female X Longitude | 0.021 | 0.357 | 0.06 | 0.95 |

**Table S4.3** – Full linear mixed model of laying date for ADCYAP1 male and female genotypes. Female age class and environmental variables were included as fixed effects and tested for interactions with breeder genotypes. Female identity, male identity and year were included as random effects and all explanatory variables were standardized. Bold variables were kept in the final model.

| Variables | Estimates | S.E. | t-value | *P*-value |
| --- | --- | --- | --- | --- |
| Intercept | **138.935** | **0.873** | **159.173** | **<0.001** |
| Age | **5.326** | **0.710** | **7.50** | **<0.001** |
| Density | **-1.238** | **0.298** | **4.16** | **<0.001** |
| April temperature | **-0.978** | **0.480** | **2.04** | **0.048** |
| Latitude | **0.372** | **0.306** | **1.22** | **0.23** |
| ADCYAP1 female | **0.103** | **0.293** | **0.35** | **0.73** |
| ADCYAP1 female X Latitude | **0.697** | **0.316** | **2.21** | **0.028** |
| ADCYAP1 male | -0.066 | 0.288 | 0.23 | 0.82 |
| Longitude | 0.025 | 0.397 | 0.06 | 0.95 |
| ADCYAP1 male X Age | -1.134 | 0.703 | 1.62 | 0.11 |
| ADCYAP1 female X April temperature | -0.396 | 0.266 | 1.49 | 0.14 |
| ADCYAP1 female X Age | -0.756 | 0.718 | 1.05 | 0.29 |
| ADCYAP1 female X Density | -0.393 | 0.316 | 1.25 | 0.21 |
| ADCYAP1 male X Latitude | 0.306 | 0.294 | 1.04 | 0.30 |
| ADCYAP1 female X ADCYAP1 male | -0.302 | 0.312 | 0.97 | 0.33 |
| ADCYAP1 male X Longitude | -0.224 | 0.299 | 0.75 | 0.45 |
| ADCYAP1 male X April temperature | -0.176 | 0.277 | 0.63 | 0.53 |
| ADCYAP1 male X Density | -0.135 | 0.292 | 0.46 | 0.64 |
| ADCYAP1 female X Longitude | 0.113 | 0.350 | 0.32 | 0.75 |

**Table S4.4** – Full linear mixed model of laying date for CREB1 male and female genotypes. Environmental variables were included as fixed effects and tested for interactions with breeder genotypes. Female age class was also included as fixed effect and female identity, male identity and year were included as random effects and all explanatory variables were standardized. Bold variables were kept in the final model.

| Variables | Estimates | S.E. | t-value | *P*-value |
| --- | --- | --- | --- | --- |
| Intercept | **138.946** | **0.865** | **160.64** | **<0.001** |
| Age | **5.383** | **0.710** | **7.58** | **<0.001** |
| Density | **-1.231** | **0.298** | **4.13** | **<0.001** |
| April temperature | **-1.001** | **0.478** | **2.10** | **0.043** |
| CREB1 male | -0.385 | 0.284 | 1.36 | 0.18 |
| CREB1 female | -0.346 | 0.304 | 1.14 | 0.26 |
| Latitude | 0.282 | 0.304 | 0.93 | 0.35 |
| Longitude | -0.044 | 0.397 | 0.11 | 0.91 |
| CREB1 male X Density | 0.360 | 0.271 | 1.33 | 0.18 |
| CREB1 female X April temperature | -0.343 | 0.291 | 1.18 | 0.24 |
| CREB1 female X Longitude | -0.253 | 0.286 | 0.88 | 0.38 |
| CREB1 female X CREB1 male | 0.193 | 0.213 | 0.91 | 0.37 |
| CREB1 male X Longitude | 0.272 | 0.306 | 0.89 | 0.38 |
| CREB1 female X Density | -0.128 | 0.363 | 0.35 | 0.72 |
| CREB1 male X Latitude | -0.104 | 0.356 | 0.29 | 0.77 |
| CREB1 male X April temperature | -0.018 | 0.290 | 0.06 | 0.95 |
| CREB1 female X Latitude | -0.009 | 0.366 | 0.03 | 0.98 |

**Table S4.5** – Full linear mixed model of incubation duration for CLOCK male and female genotypes. Female age class and environmental variables were included as fixed effects and tested for interactions with breeder genotypes. Female identity, male identity and year were included as random effects and all explanatory variables were standardized. Bold variables were kept in the final model.

| Variables | Estimates | S.E. | t-value | *P*-value |
| --- | --- | --- | --- | --- |
| Intercept | **11.326** | **0.363** | **31.21** | **<0.001** |
| Age | **0.318** | **0.161** | **1.97** | **0.049** |
| Longitude | 0.064 | 0.067 | 0.97 | 0.33 |
| CLOCK female | -0.047 | 0.066 | 0.71 | 0.48 |
| CLOCK male | -0.032 | 0.064 | 0.49 | 0.62 |
| May temperature | -4.7^-04^ | 0.132 | 0.00 | 1.00 |
| CLOCK male X May temperature | 0.115 | 0.065 | 1.77 | 0.078 |
| CLOCK male X Longitude | 0.142 | 0.075 | 1.91 | 0.057 |
| CLOCK female X Age | -0.196 | 0.159 | 1.23 | 0.22 |
| CLOCK female X Longitude | -0.050 | 0.075 | 0.68 | 0.50 |
| CLOCK female X May temperature | -0.058 | 0.072 | 0.81 | 0.42 |
| CLOCK female X CLOCK male | -0.025 | 0.059 | 0.42 | 0.67 |
| CLOCK male X Age | -0.023 | 0.140 | 0.17 | 0.88 |

**Table S4.6** – Full linear mixed models of incubation duration for NPAS1 male and female genotypes. Female age class and environmental variables were included as fixed effects and tested for interactions with breeder genotypes. Female identity, male identity and year were included as random effects and all explanatory variables were standardized. Bold variables were kept in the final model.

| Variables | Estimates | S.E. | t-value | *P*-value |
| --- | --- | --- | --- | --- |
| Intercept | **11.323** | **0.360** | **31.43** | **<0.001** |
| Age | **0.332** | **0.160** | **2.08** | **0.039** |
| NPAS2 male | **0.144** | **0.063** | **2.28** | **0.023** |
| Longitude | 0.073 | 0.067 | 1.09 | 0.28 |
| NPAS2 female | 0.021 | 0.066 | 0.32 | 0.75 |
| May temperature | 0.009 | 0.131 | 0.07 | 0.95 |
| NPAS2 male X May temperature | 0.113 | 0.064 | 1.78 | 0.076 |
| NPAS2 female X Age | -0.249 | 0.159 | 1.56 | 0.12 |
| NPAS2 male X Longitude | -0.040 | 0.073 | 0.55 | 0.58 |
| NPAS2 male X Age | 0.076 | 0.163 | 0.49 | 0.64 |
| NPAS2 female X NPAS2 male | 0.016 | 0.074 | 0.22 | 0.83 |
| NPAS2 female X May temperature | -0.006 | 0.063 | 0.09 | 0.93 |
| NPAS2 female X Longitude | -0.009 | 0.078 | 0.11 | 0.91 |

**Table S4.7** – Full linear mixed model of incubation duration for ADCYAP1 male and female genotypes. Female age class and environmental variables were included as fixed effects and tested for interactions with breeder genotypes. Female identity, male identity and year were included as random effects and all explanatory variables were standardized. Bold variables were kept in the final model.

| Variables | Estimates | S.E. | t-value | *P*-value |
| --- | --- | --- | --- | --- |
| Intercept | **11.326** | **0.363** | **31.21** | **<0.001** |
| Age | **0.318** | **0.161** | **1.97** | **0.049** |
| ADCYAP1 female | 0.071 | 0.065 | 1.10 | 0.27 |
| Longitude | 0.067 | 0.064 | 1.00 | 0.32 |
| ADCYAP1 male | 0.032 | 0.064 | 0.51 | 0.61 |
| May temperature | -0.021 | 0.131 | 0.16 | 0.88 |
| ADCYAP1 female X Longitude | -0.081 | 0.071 | 1.13 | 0.26 |
| ADCYAP1 male X Age | -0.147 | 0.161 | 0.91 | 0.36 |
| ADCYAP1 female X Age | -0.107 | 0.162 | 0.55 | 0.51 |
| ADCYAP1 female X ADCYAP1 male | 0.038 | 0.071 | 0.54 | 0.59 |
| ADCYAP1 male X May temperature | -0.019 | 0.059 | 0.32 | 0.75 |
| ADCYAP1 male X Longitude | -0.030 | 0.074 | 0.40 | 0.69 |
| ADCYAP1 female X May temperature | -0.003 | 0.068 | 0.04 | 0.97 |

**Table S4.8** – Full linear mixed model of incubation duration for CREB1 male and female genotypes. Environmental variables were included as fixed effects and tested for interactions with breeder genotypes. Female age class was also included as fixed effect. Female identity, male identity and year were included as random effects and all explanatory variables were standardized. Bold variables were kept in the final model.

| Variables | Estimates | S.E. | t-value | *P*-value |
| --- | --- | --- | --- | --- |
| Intercept | **11.302** | **0.315** | **35.89** | **<0.001** |
| Age | **0.322** | **0.159** | **2.02** | **0.044** |
| May temperature | **-0.097** | **0.094** | **1.03** | **0.30** |
| CREB1 male | **-0.108** | **0.062** | **1.73** | **0.088** |
| CREB1 male X May temperature | **0.217** | **0.058** | **3.73** | **<0.001** |
| Longitude | 0.044 | 0.088 | 0.50 | 0.62 |
| CREB1 female | -0.004 | 0.066 | 0.06 | 0.95 |
| CREB1 female X Longitude | 0.065 | 0.057 | 1.14 | 0.26 |
| CREB1 female X May temperature | 0.119 | 0.072 | 1.66 | 0.097 |
| CREB1 male X Longitude | 0.051 | 0.074 | 0.69 | 0.49 |
| CREB1 female X CREB1 male | 0.017 | 0.049 | 0.34 | 0.73 |

***S5.*** *Supplementary information on allelic diversity*

**Table S5.1** – Summary of studies assessing allelic diversity for CLOCK, NPAS2, ADCYAP1 and CREB1 in various bird species. Number of observed alleles (N alleles) and observed heterozygosity (*Ho*) are reported.

| Species | Localization | Candidate gene | N alleles | *Ho* | Reference |
| --- | --- | --- | --- | --- | --- |
| Barn swallow (*Hirundo rustica*) | Milano, Italy | CLOCK | 4 | 0.066 | Caprioli *et al.* 2012 |
| Barn swallow (*Hirundo rustica*) | Worldwild (5 sites) | CLOCK | 3 | 0.030 | Dor *et al.* 2011 |
| Barn swallow (*Hirundo rustica*) | Boje, Nigeria | CLOCK | 3 | - | Saino *et al.* 2013 |
| Blackcaps (*Sylvia atricapilla*) | Worldwild (14 sites) | CLOCK | 8 | - | Mueller *et al.* 2011 |
| Blue tit (*Cyanistes caeruleus*) | Worldwild (14 sites) | CLOCK | 9 | 0.489 | Johnsen *et al.* 2007 |
| Blue tit (*Cyanistes caeruleus*) | Wytham Woods, Oxfordshire, UK | CLOCK | 6 | 0.565 | Liedvogel *et al.* 2009 |
| Blue tit (*Cyanistes caeruleus*) | Wytham Woods, Oxfordshire, UK | CLOCK | 5 | 0.561 | Olano-Marin *et al.* 2011 |
| Blue tit (*Cyanistes caeruleus*) | Wytham Woods, Oxfordshire, UK | CLOCK | 5 | 0.57 | Steinmeyer *et al.* 2009 |
| Blue tit (*Cyanistes caeruleus*) | Wytham Woods, Oxfordshire, UK | CLOCK | 4 | 0.60 | Steinmeyer *et al.* 2012 |
| Bluethroat (*Luscinia svecica*) | Worldwild (12 sites) | CLOCK | 7 | 0.213 | Johnsen *et al.* 2007 |
| Common buzzard (*Buteo buteo*) | Eastern Westphalia, Germany | CLOCK | 1 | - | Chakarov *et al.* 2013 |
| Great tit (*Parus major*) | Wytham Woods, Oxfordshire, UK | CLOCK | 5 | 0.077 | Liedvodel & Sheldon 2010 |
| Junco spp. (2 species) | Americas (15 sites) | CLOCK | 8 | 0.294 | Peterson *et al.* 2013 |
| Pied flycatcher (*Ficedula hypoleuca*) | La Hiruela, Spain | CLOCK | 5 | 0.722 | Kuhn *et al.* 2013 |
| Raptors (10 species) | Adlerwarte Berlebeck, Germany | CLOCK | 2 | - | Chakarov *et al.* 2013 |
| *Tachycineta spp.* (5 species) | Americas (5 sites) | CLOCK | 5 | 0.332 | Dor *et al.* 2012 |
| Blackcaps (*Sylvia atricapilla*) | Worldwild (14 sites) | NPAS2 | 2 | - | Mueller *et al.* 2011 |
| Blue tit (*Cyanistes caeruleus*) | Wytham Woods, Oxfordshire, UK | NPAS2 | 8 | 0.742 | Olano-Marin *et al.* 2011 |
| Blue tit (*Cyanistes caeruleus*) | Wytham Woods, Oxfordshire, UK | NPAS2 | 5 | 0.75 | Steinmeyer *et al.* 2009 |
| Blue tit (*Cyanistes caeruleus*) | Wytham Woods, Oxfordshire, UK | NPAS2 | 6 | 0.75 | Steinmeyer *et al.* 2012 |
| Common buzzard (*Buteo buteo*) | Eastern Westphalia, Germany | NPAS2 | 2 | 0.014 | Chakarov *et al.* 2013 |
| Raptors (10 species) | Adlerwarte Berlebeck, Germany | NPAS2 | 2 | - | Chakarov *et al.* 2013 |
| Blackcaps (*Sylvia atricapilla*) | Worldwild (14 sites) | ADCYAP1 | 13 | - | Mueller *et al.* 2011 |
| Blue tit (*Cyanistes caeruleus*) | Wytham Woods, Oxfordshire, UK | ADCYAP1 | 14 | 0.637 | Olano-Marin *et al.* 2011 |
| Blue tit (*Cyanistes caeruleus*) | Wytham Woods, Oxfordshire, UK | ADCYAP1 | 7 | 0.68 | Steinmeyer *et al.* 2009 |
| Blue tit (*Cyanistes caeruleus*) | Wytham Woods, Oxfordshire, UK | ADCYAP1 | 9 | 0.64 | Steinmeyer *et al.* 2012 |
| Common buzzard (*Buteo buteo*) | Eastern Westphalia, Germany | ADCYAP1 | 3 | 0.312 | Chakarov *et al.* 2013 |
| *Junco* *spp.* (2 species) | Americas (15 sites) | ADCYAP1 | 16 | 0.772 | Peterson *et al.* 2013 |
| Raptors (10 species) | Adlerwarte Berlebeck, Germany | ADCYAP1 | 6 | - | Chakarov *et al.* 2013 |
| Blackcaps (*Sylvia atricapilla*) | Worldwild (14 sites) | CREB1 | 10 | - | Mueller *et al.* 2011 |
| Blue tit (*Cyanistes caeruleus*) | Wytham Woods, Oxfordshire, UK | CREB1 | 9 | 0.267 | Olano-Marin *et al.* 2011 |
| Blue tit (*Cyanistes caeruleus*) | Wytham Woods, Oxfordshire, UK | CREB1 | 7 | 0.27 | Steinmeyer *et al.* 2009 |
| Blue tit (*Cyanistes caeruleus*) | Wytham Woods, Oxfordshire, UK | CREB1 | 6 | 0.30 | Steinmeyer *et al.* 2012 |
| Common buzzard (*Buteo buteo*) | Eastern Westphalia, Germany | CREB1 | 3 | 0.093 | Chakarov *et al.* 2013 |
| Raptors (10 species) | Adlerwarte Berlebeck, Germany | CREB1 | 2 | - | Chakarov *et al.* 2013 |

***S6.*** *References*

Bourret A, Bélisle M, Pelletier F, Garant D (submitted) Multidimensional environmental influences on timing of breeding in a tree swallow population facing climate change.

Caprioli M, Ambrosini R, Boncoraglio G *et al.* (2012) Clock gene variation is associated with breeding phenology and maybe under directional selection in the migratory barn swallow. *Plos One*, **7,** e35140.

Chakarov N, Jonker RM, Boerner M, Hoffman JI, Krüger O (2013) Variation at phenological candidate genes correlates with timing of dispersal and plumage morph in a sedentary bird of prey. *Molecular Ecology*, **22**, 5430–5440.

Dor R, Cooper CB, Lovette IJ *et al.* (2012) Clock gene variation in *Tachycineta* swallows. *Ecology and evolution*, **2**, 95–105.

Dor R, Lovette IJ, Safran RJ *et al.* (2011) Low variation in the polymorphic clock gene poly-Q region despite population genetic structure across barn swallow (*Hirundo rustica*) populations. *Plos One*, **6**, e28843.

Johnsen A, Fidler AE, Kuhn S *et al.* (2007) Avian clock gene polymorphism: Evidence for a latitudinal cline in allele frequencies. *Molecular Ecology*, **16**, 4867–4880.

Kuhn K, Schwenk K, Both C *et al.* Differentiation in neutral genes and a candidate gene in the pied flycatcher: Using biological archives to track global climate change. *Ecology and Evolution*, **3**, 4799–4814.

Liedvogel M, Sheldon BC (2010) Low variability and absence of phenotypic correlates of Clock gene variation in a great tit *Parus major* population. *Journal of Avian Biology*, **41**, 543–550.

Liedvogel M, Szulkin M, Knowles SCL, Wood MJ, Sheldon BC (2009) Phenotypic correlates of Clock gene variation in a wild blue tit population: Evidence for a role in seasonal timing of reproduction. *Molecular ecology*, **18**, 2444–56.

Mueller JC, Pulido F, Kempenaers B (2011) Identification of a gene associated with avian migratory behaviour. *Proceedings of the Royal Society B: Biological Sciences*, **278**, 2848–2856.

Nakagawa S, Schielzeth H (2010) Repeatability for Gaussian and non-Gaussian data: a practical guide for biologists. *Biological Reviews*, **85**, 935–956.

Olano-Marin J, Mueller JC, Kempenaers B (2011) Heterozygosity and survival in blue tits (*Cyanistes caeruleus*): contrasting effects of presumably functional and neutral loci. *Molecular Ecology*, **20**, 4028–4041.

Peterson MP, Abolins-Abols M, Atwell JW *et al.* (2013) Variation in candidate genes CLOCK and ADCYAP1 does not consistently predict differences in migratory behavior in the songbird genus Junco [v1; ref status: indexed, http://f1000r.es/11p]. *F1000Research 2013*, **2**, 115.

Saino N, Romano M, Caprioli M *et al.* (2013) Timing of molt of barn swallows is delayed in a rare Clock genotype. *PeerJ*, **1**, e17.

Steinmeyer C, Kempenaers B, Mueller JC (2012) Testing for associations between candidate genes for circadian rhythms and individual variation in sleep behaviour in blue tits. *Genetica*, **140**, 219–228.

Steinmeyer C, Mueller JC, Kempenaers B (2009) Search for informative polymorphisms in candidate genes: Clock genes and circadian behaviour in blue tits. *Genetica*, **136**, 109–117.
